# Clinical presentation and microbial culture among osteomyelitis patients

**DOI:** 10.6026/973206300210309

**Published:** 2025-03-31

**Authors:** Babita Kumari, Rashmi Prabha, Vijay Kumar

**Affiliations:** 1 Department of Microbiology, Patna Medical College, Patna, Bihar, India

**Keywords:** Osteomyelitis, clinical presentation, microbial culture

## Abstract

Clinical presentation and microbial culture among osteomyelitis patients is required for proper diagnosis and management. Therefore, it is of interest to evaluate the clinical presentation and microbial culture among osteomyelitis patients. Hence, 200 patients with osteomyelitis having clinical symptoms and radiological findings were qualified for participation. Specimens such as synovial fluid, bone sequestrum, pus swabs and pus were collected aseptically and examined for microbial growth. Clinical assessment of osteomyelitis patient showed that most commonly affected bone was tibia with trauma. Inability to bear weight was commonly observed with symptoms like fever, pain, or tenderness and swelling where infection is the predisposing factor for osteomyelitis. Further, different microorganisms like *Staphylococcus aureus*, *Escherichia coli*, *Klebsiella spp*, *etc*. were found in microbial culture.

## Background:

Osteomyelitis represents an extensive illness of the bones caused by infection. The clinical identification of osteomyelitis is usually supported by imaging as well as lab data [1, 2- 3] and bone biopsy along with culture of microbes offer definitive diagnoses. The initial course of therapy should consist of antibiotics that should be selected based on each person's distinctive features and the results of the culture [4, 5-6]. Often, bone debridement intervention is needed and in individuals who are at elevated risk or who are very unwell, further surgery may be required [7, 8-9]. Thanks to improvements in surgical expertise, administration of antibiotics and the available resources for accurate diagnosis and customized management for every type of osteomyelitis, more favourable outcomes are being achieved in the medical management of this painful condition [10, 11-12]. Communicable infection, direct infectious agent inoculation, or spread of microorganisms through bloodstream is the causes of osteomyelitis [13, 14-15]. Recent developments in the osteomyelitis epidemiological research, pathophysiology, management, diagnosis and outcome have raised interest in this ailment [14, 15-16]. It may stay localized or affect many structures, including the periosteum, cortex, bone marrow and portions of the adjacent soft tissues [17, 18]. Osteomyelitis is more prevalent in the lower extremities at the distal portion of the tibia bone and metaphysis region of the femur bone and it primarily impacts the developing endpoints of the longer bones [19, 20- 21]. Numerous microbes can enter the bloodstream and inflame bone tissues; in uncommon circumstances, soft tissue infections can result in bone injury. Via blood circulation from wounds on the skin, infections of the upper respiratory tract, periodontal disease and other pathogenic regions, microorganisms can reach the metaphysis region of bone [22, 23]. The sluggish blood flow and abundance of circulation blood vessels in the bone, metaphysic region may assist in the dissemination of infection. Osteomyelitis can arise from direct trauma to the bone [21-23]. The identification of this illness is mostly based on radiographic observations of translucency of bone with scattered sclerosis and surrounding periosteal bone response, as well as considerable clinical indications of non-healing wound, particularly in diabetic patients [25, 26]. The cornerstones of a treatment regimen for such individuals include pus culture, invasive bone biopsy, blood culture and MRI. Patients having diabetes mellitus who have foot ulcers typically have infections with numerous organisms, but the majority of infections are monomicrobial [20- 23]. More rapid as well as accurate microbiological testing methods are needed for osteomyelitis, especially for microorganisms and in the typical situation of antibiotics being administered before sampling, according to recent investigations [12-14]. Even with the advancement of better microbiological techniques in recent years, the root cause of osteomyelitis remains poorly understood [15- 17]. There are not many published contemporary studies that have well-defined populations of patients, proper gathering of specimens before antibiotic therapy and surgical debridement and comprehensive microbial diagnosis [15-18]. Therefore, it is of interest to evaluate clinical presentation and microbial culture among osteomyelitis patients.

## Methods and Materials:

200 patients who arrived at participating institutions' emergency rooms or outpatient clinics with osteomyelitis as clinical and radiological diagnosis were qualified for participation and evaluated for consideration in the study. The radiographic findings of bone translucency with scattered sclerosis and the surrounding periosteal bone response served as the primary basis for the diagnosis of this disease. Significant clinical signs of non-healing wounds, especially in people with diabetes. MRI, blood culture, invasive bone biopsy and pus culture are the mainstays of a therapeutic plan for these patients.

## Inclusion criteria:

The study comprised clinically and radiologically confirmed cases of osteomyelitis across both genders and across all age categories. Specimen such as synovial fluid (SF), bone sequestrum, pus swabs and pus were collected aseptically and examined for responsiveness and growth.

## Exclusion criteria:

The study eliminated participants with osteomyelitis who were receiving antibiotic treatment, patients with history of old trauma, with non-union bones, patients with no history of infection, patient with cysts, malignant tumours and benign tumours.

## Sample collection and preliminary identification by biochemical tests:

A sterilized vessel was used for gathering all clinical samples, surgically excised tissue, bone sequestrum and pus specimens that had been collected from the patient. Then, using normal techniques (biochemical testing and Gram staining), the initial detection was completed. Gram stain anatomy, colony characteristics and biochemical processes were used to identify the culture specimens.

## Statistical analysis:

Results were presented as percentages and frequencies once the data was imported into Microsoft Excel. Group variations among categorical parameters were evaluated using either Fisher's exact test or chi-square test. One-way analysis of variance (ANOVA) was applied to continuous variables. The threshold for statistical significance was p < 0.05. Every probability was two-tailed. SPSS software (version 15.0, SPSS Inc., Chicago, IL, USA) was used for all statistical analyses.

## Results and Discussion:

Most commonly affected bone was Tibia being affected in 101 (50.5%) osteomyelitis patients followed by Femur being affected in 72 (36.0%) osteomyelitis patients (Table 1). Trauma was the most predisposing factors for osteomyelitis being observed in 97 (48.5%) patients. It was followed by orthopaedic implants being observed in 37 (18.5%) patients. Other predisposing factors were postoperative infection and Implant/Diabetes mellitus being observed in 19 (9.5%) patients and 7 (3.5%) patients respectively (Table 2). Different symptoms of osteomyelitis were fever being observed in 137 (68.5%) osteomyelitis patients, pain, or tenderness in 181 (90.5%) osteomyelitis patients, swelling in 174 (87.0%) osteomyelitis patients, Inability to weight bear in 91 (45.5%) patients and joint immobility in 135 (67.5%) patients (Table 3). 154 (77%) osteomyelitis cases were considered as acute cases in which mean number of days between detection of symptoms and diagnosis of osteomyelitis was 5.9+ 3.6 days while 46 (23%) osteomyelitis cases were considered as subacute with mean number of days between detection of symptoms and diagnosis of osteomyelitis was 26.4 + 5.3 days (Table 4). The most prevalent microorganisms detected in osteomyelitis specimens were *Staphylococcus aureus* being detected in 96 (48%) specimens followed by *Escherichia coli* being detected in 29 (14.5%) patients, *Klebsiella spp* in 24 (12%) patients. Other microorganisms detected were Pseudomonas spp detected in 19 (9.5%) patients, Proteus spp. detected in 11 (5.5%) cases (Table 5).

An infection-related, widespread bone disease is called osteomyelitis. Imaging and laboratory evidence typically corroborate the clinical diagnosis of osteomyelitis. Microbe culture and bone biopsy provide conclusive diagnosis [21- 23]. This study was conducted with aim of evaluating clinical presentation and detecting microbial culture among osteomyelitis patients. In our study, most commonly affected bone was Tibia being affected in 101 (50.5%) osteomyelitis patients followed by Femur being affected in 72 (36.0%) osteomyelitis patients. Trauma was the most predisposing factors for osteomyelitis being observed in 97 (48.5%) patients. It was followed by orthopaedic implants being observed in 37 (18.5%) patients. Other predisposing factors were postoperative infection and Implant/Diabetes mellitus being observed in 19 (9.5%) patients and 7 (3.5%) patients respectively. The above findings of our study have similarity with the findings of other studies [20- 23]. These studies like our study found that long bones like tibia are most commonly affected bones in osteomyelitis [21-24]. Some studies like our study showed that trauma and infection constitute the major proportion of predisposing factors for osteomyelitis [23- 25]. Osteomyelitis is caused by a communicable infection, direct inoculation with an infectious agent, or the transfer of bacteria through the circulation [20, 21, 22-23]. Interest in osteomyelitis has increased due to recent advancements in epidemiological research, pathogenesis, therapy, diagnosis and outcome [14, 15]. Numerous structures, including as the periosteum, cortex, bone marrow and parts of the surrounding soft tissues, may be affected, or it may remain confined [13-17]. Several studies has stated that osteomyelitis mostly affects the developing endpoints of the longer bones and is more common in the lower extremities at the metaphysis region of the femur bone and the distal part of the tibia bone [20- 23]. Many microorganisms can infiltrate the bloodstream and cause inflammation of bone tissues; in rare cases, soft tissue infections can cause bone damage [21-24]. Microorganisms can enter the metaphysis region of bone through blood circulation from wounds on the skin, upper respiratory tract infections, periodontal disease and other pathogenic regions [19-23]. Infection may spread more easily in the bone, metaphysic region due to the slow blood flow and large number of circulation blood vessels. Direct trauma to the bone can result in osteomyelitis [15-17]. In our study, different symptoms of osteomyelitis were fever being observed in 137 (68.5%) osteomyelitis patients, pain, or tenderness in 181 (90.5%) osteomyelitis patients, swelling in 174 (87.0%) osteomyelitis patients, Inability to weight bear in 91 (45.5%) patients and joint immobility in 135 (67.5%) patients. 154 (77%) osteomyelitis cases were considered as acute cases in which mean number of days between detection of symptoms and diagnosis of osteomyelitis was 5.9+ 3.6 days while 46 (23%) osteomyelitis cases were considered as subacute with mean number of days between detection of symptoms and diagnosis of osteomyelitis was 26.4 + 5.3 days. The findings of our study are having similarity with the findings of other studies that also found symptoms like fever, pain, or tenderness, swelling, inability to bear weight [25, 26]. Like our study other studies also observed that most of the cases of osteomyelitis are acute and some cases are subacute [21- 24]. The microbial assessment of specimens in our study revealed that the most prevalent microorganisms detected in osteomyelitis specimens were *Staphylococcus aureus* being detected in 96(48%) specimens followed by *Escherichia coli* being detected in 29 (14.5%) patients, *Klebsiella spp* in 24 (12%) patients. Other microorganisms detected were Pseudomonas spp detected in 19 (9.5%) patients, Proteus spp. detected in 11 (5.5%) cases. The results of our study are having resemblance with the findings of other studies that also found different microorganisms like *Staphylococcus aureus*, being isolated from culture specimens *Escherichia coli*, *Klebsiella spp* others [17-21]. According to studies, osteomyelitis requires more precise and quick microbiological testing techniques, particularly for microorganisms and in the common scenario of antibiotics being given prior to sample [17-19]. The underlying cause of osteomyelitis is still not well known, despite recent improvements in microbiological techniques [13-16]. Few studies have been published with clearly characterized patient groups, appropriate specimen collection prior to antibiotic treatment and surgical debridement and thorough microbiological diagnosis [17-20].

## Conclusion:

Clinical assessment of osteomyelitis patient showed that most commonly affected bone was tibia with trauma. Inability to bear weight was commonly observed with symptoms like fever, pain, or tenderness and swelling where infection is the predisposing factor for osteomyelitis. Further, different microorganisms like *Staphylococcus aureus*, *Escherichia coli*, *Klebsiella spp* *etc*. were found in microbial culture.

## Supplementary material:

No

## Author contribution:

Each author has made a substantial contribution to the conception or design of the work, acquisition, analysis and interpretation of data and has drafted the work and substantively revised it.

## Funding:

This research received no external funding.

## Figures and Tables

**Table 1:** Involvement of different bones in osteomyelitis

	**Tibia**	**Femur**	**Fibula**	**Ulna**	**Radius**	**Metacarpal**	**Metatarsal**	**Humerus**	**Calcaneus**
No	101	72	7	5	3	3	3	3	3
%	50.5	36	3.5	2.5	1.5	1.5	1.5	1.5	1.5

**Table 2:** Different predisposing variables for osteomyelitis

	**Trauma**	**Orthopaedic implants**	**Postoperative infection**	**Implant/Diabetes mellitus**	**Postoperative infection/Diabetes mellitus**	**Trauma/Diabetes mellitus**
No	97	37	19	7	3	3
%	48.5	18.5	9.5	3.5	1.5	1.5

**Table 3:** Symptoms of osteomyelitis

	**Fever**	**Pain or tenderness**	**Swelling**	**Inability to weight bear**	**Joint immobility**
No	137	181	174	91	135
%	68.5	90.5	87	45.5	67.5

**Table 4:** Number of days between detection of symptoms and diagnosis of osteomyelitis

	**Acute cases**	**Sub-acute cases**
No	154	46
%	77	23
Number of days between detection of symptoms and diagnosis of osteomyelitis (mean ± SD)	5.9+3.6	26.4+5.3

**Table 5:** Different micro-organisms detected in osteomyelitis specimens

	**Staphylococcus aureus**	**Staphylococcus lugdunensis**	**CoNS**	**Escherichia coli**	**Klebsiella spp.**	**Pseudomonas spp.**	**Proteus spp.**	**Acinetobacter baumanni**
No	96	7	7	29	24	19	11	7
%	48	3.5	3.5	14.5	12	9.5	5.5	3.5
